# Bayesian cost-effectiveness analysis of Whole genome sequencing versus Whole exome sequencing in a pediatric population with suspected genetic disorders

**DOI:** 10.1007/s10198-023-01644-0

**Published:** 2023-11-17

**Authors:** Mario Cesare Nurchis, Francesca Clementina Radio, Luca Salmasi, Aurora Heidar Alizadeh, Gian Marco Raspolini, Gerardo Altamura, Marco Tartaglia, Bruno Dallapiccola, Gianfranco Damiani

**Affiliations:** 1 School of Economics, Università Cattolica del Sacro Cuore, Largo Francesco Vito 1, 00168 Rome, Italy; 2 Department of Woman and Child Health and Public Health, Fondazione Policlinico Universitario A. Gemelli IRCCS, 00168 Rome, Italy; 3 Molecular Genetics and Functional Genomics, Ospedale Pediatrico Bambino Gesù IRCCS, 00146 Rome, Italy; 4 Department of Economics and Finance, Università Cattolica del Sacro Cuore, 00168 Rome, Italy; 5 Department of Health Sciences and Public Health, Section of Hygiene, Università Cattolica del Sacro Cuore, 00168 Rome, Italy

**Keywords:** Pediatric population, Genomic sequencing, Exome sequencing, Bayesian cost-effectiveness analysis, Public Health, C11, C80, I18

## Abstract

Genetic diseases are medical conditions caused by sequence or structural changes in an individual’s genome. Whole exome sequencing (WES) and whole genome sequencing (WGS) are increasingly used for diagnosing suspected genetic conditions in children to reduce the diagnostic delay and accelerating the implementation of appropriate treatments. While more information is becoming available on clinical efficacy and economic sustainability of WES, the broad implementation of WGS is still hindered by higher complexity and economic issues. The aim of this study is to estimate the cost-effectiveness of WGS versus WES and standard testing for pediatric patients with suspected genetic disorders. A Bayesian decision tree model was set up. Model parameters were retrieved both from hospital administrative datasets and scientific literature. The analysis considered a lifetime time frame and adopted the perspective of the Italian National Health Service (NHS). Bayesian inference was performed using the Markov Chain Monte Carlo simulation method. Uncertainty was explored through a probabilistic sensitivity analysis (PSA) and a value of information analysis (VOI). The present analysis showed that implementing first-line WGS would be a cost-effective strategy, against the majority of the other tested alternatives at a threshold of €30,000–50,000, for diagnosing outpatient pediatric patients with suspected genetic disorders. According to the sensitivity analyses, the findings were robust to most assumption and parameter uncertainty. Lessons learnt from this modeling study reinforces the adoption of first-line WGS, as a cost-effective strategy, depending on actual difficulties for the NHS to properly allocate limited resources.

**Supplementary Information:**

The online version contains supplementary material available at 10.1007/s10198-023-01644-0.

## Introduction

Genetic diseases are medical conditions caused by sequence or structural changes in an individual’s genome [1]. Genetic pediatric disorders manifest either prenatally or appear at birth or in childhood and represent a significant clinical and economic burden. These conditions, which often are rare diseases (RDs), result in overall health care expenses ranging from $4.6 to 17.5 billion, which accounts for roughly 12–47% of the national expenditure on inpatient medical care for children in the United States of America [2]. In 2019, the total economic burden of RDs in United States was calculated in $966 billion, including $548 billion (57%) of indirect and non-medical costs and $418 billion (43%) of direct medical costs. Genetic disorders, in particular RDs, might be difficult to diagnose due to non-specific or poorly characterized clinical manifestations, exacerbating their burden [3]. In standard diagnostics, the sequential use of single-gene analysis was frequently involved. However, for specific highly heterogeneous clinical conditions, multi-gene panels and/or genome-wide sequencing approaches are used to search for the underlying causative variation(s). Due to the high number of disease genes, the different forms of pathogenic variations, and heterogeneity of RDs [4], diagnosis of RDs is challenging, lengthy, and might involve multiple iterations [5]. As a result, patients with suspected genetic diseases often experience a diagnostic odyssey, with long periods of uncertainty, leading to increased health and financial burdens, such as missed opportunities for timely intervention, unnecessary procedures, treatments, specialist visits, and significant emotional and financial problems for their families [6, 7].

Next-generation sequencing (NGS) technologies have enabled the diagnosis of genetic diseases by enhancing the capacity to sequence larger segments of the genome [8]. While NGS technologies can be used to examine individual genes or groups of genes, whole exome sequencing (WES) allows the analysis of the protein-coding sections of the genome, and whole genome sequencing (WGS) of both the coding and noncoding regions. WES and WGS are increasingly used for diagnosing suspected genetic conditions in children, with the goal of reducing the diagnostic delay and accelerating the implementation of appropriate treatments. It has been shown that WES and WGS increase effectiveness in detecting genetic diseases and have higher rates of clinical utility [9–11].

While more information is becoming available on clinical efficacy and economic sustainability of WES, the broad implementation of WGS is still hindered by higher complexity and economic issues. Economic evaluations could steer the decision-making process in providing access to WES and WGS to suitable pediatric patients by taking into account the associated costs, which is crucial for the health systems sustainability. Nowadays, there are a number of significant studies that have examined the cost-effectiveness of WES [12]. Nonetheless, up to date, only few studies examined the cost-effectiveness of both WGS and WES in the pediatric population with suspected genetic disorders [13–15], highlighting promising results. The aim of this study is to estimate the cost-effectiveness of WGS versus WES and standard testing for pediatric patients with suspected genetic disorders.

## Methods

### Framing the model

#### Target population

The present modeling study was based on a cohort of 870 pediatric patients with suspected genetic disorders. The standard of care (SOC) option involved a group of 300 patients while WES and WGS a group of 480 and 90 patients, respectively.

The target population consisted of undiagnosed pediatric patients facing potentially life-threatening illnesses. This cohort included only patients whose first access to the hospital was in an outpatient setting, and who received monitoring and evaluation as part of their outpatient care. These patients were a matter of significant concern due to their vulnerability to potentially fatal complications, even if they were not hospitalized during the time of data collection. Clinical presentations and medical histories were heterogeneous, but typically included encephalopathies, epilepsies, neuromuscular disorders, intellectual disability with dysmorphic features, and other non-specific presentations of suspected genetic origin.

The model excluded patients with genetic conditions diagnosed through prenatal (e.g., trisomy 21) or newborn screening (e.g., cystic fibrosis) and disorders for which clear clinical criteria were established and single-gene testing was preferred (e.g., neurofibromatosis).

The selected population reflects the one enrolled in one of the largest pediatric hospitals in Europe, recognized for all subspecialties.

#### Study perspective, setting, and location

The cost-effectiveness analysis was conducted according to the Italian National Health Service’s (NHS) perspective.

#### Intervention and comparators

The cost-effectiveness analysis involved the comparison between WGS and other four comparators in line with the testing strategies available today. The comparators were SOC testing only; first-line WES; SOC followed by WES; and SOC followed by WGS. For the purposes of this study, SOC refers to the combination of standard genetic tests and diagnostic investigations, typically included in routine clinical practice, involving single-gene panels, multi-gene panels, chromosomal microarray (CMA), and karyotype while WES is not included as part of the standard diagnostic workup. The intervention was based on the WGS.

#### Time horizon, discount rate, and threshold

A lifetime time horizon was set. In line with the NICE recommendations [16], we hypothesized that the chosen time horizon is adequate to assess the benefits of the intervention. Both costs and effects were discounted by a 3% yearly rate. In relation to the threshold choice, it was necessary to steer the decision-making process on which alternative to support, the Eurozone threshold, ranging from €30,000 to €50,000, was adopted.

### Model structure

#### Type of model

**Fig. 1:**
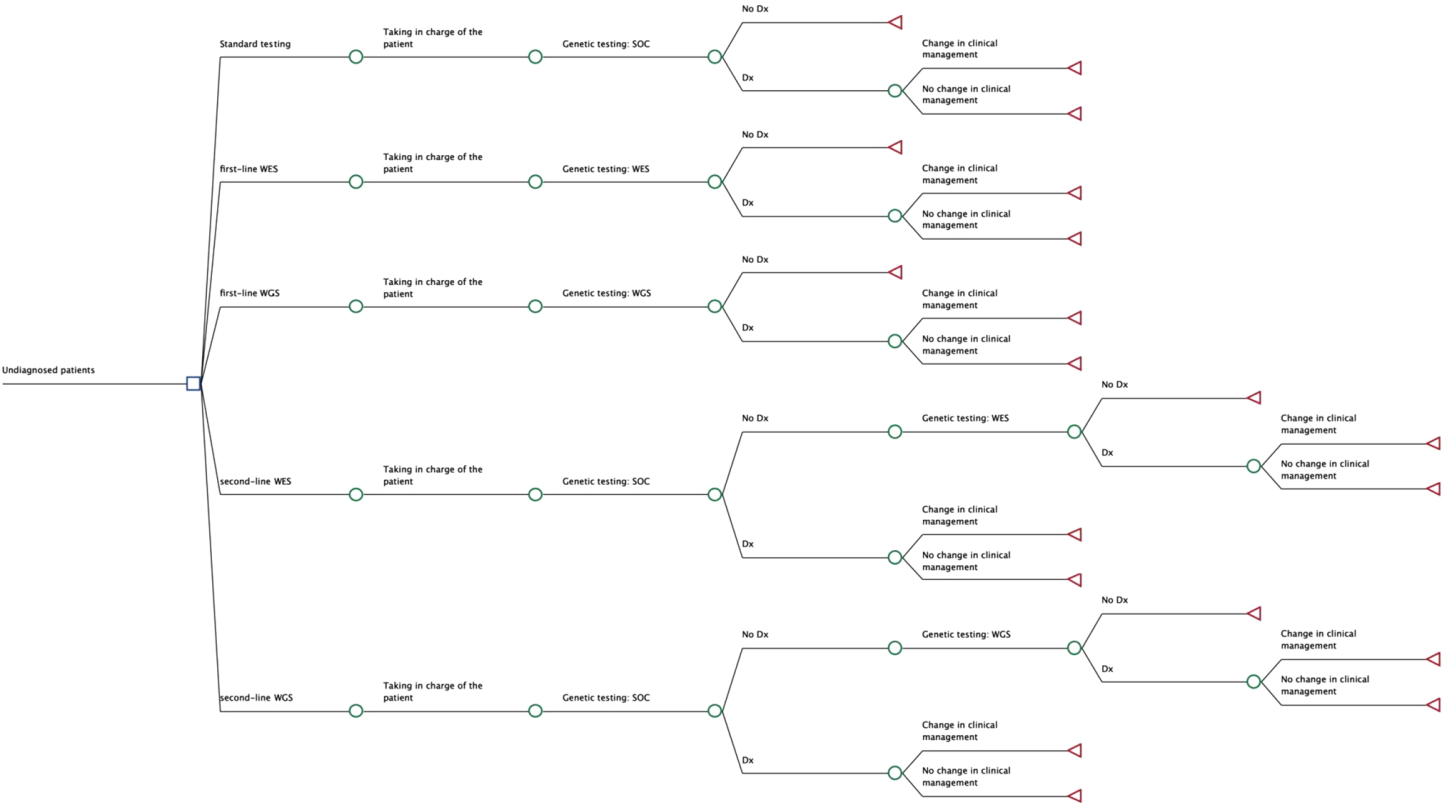
Decision tree model structure

A Bayesian decision tree model was set up. As depicted in Fig. 1, the model included five arms, one for each strategy.

After the patient’s taking charge by a clinical geneticist, they undergo the arm-specific genetic sequencing test. After this stage, the patient could achieve a definitive diagnosis or not based on determined probabilities. In case of a multi-step process, in absence of diagnosis, the patient undergoes a second genetic test. Lastly, when a diagnosis is achieved, the patient may experience or not a change in clinical management.

A Bayesian model defines a full probability distribution of a bivariate outcome, $\theta =(c,e)$ comprising possible combinations of costs and effects, to allow researchers to: (*i*) perform the decision analysis on average values, and (*ii*) perform the probabilistic sensitivity analysis to understand the uncertainty surrounding the decision. According to the Bayes theorem, we can write:

$$p\left(\theta |D\right)=\frac{p(D|\theta )p(\theta )}{p(D)}$$

$p\left(\theta |D\right)$ is the posterior, i.e., the credibility of parameters given the available data ($D$); $p(\theta )$ is the prior for the parameter $\theta$, i.e., the credibility of the $\theta$ values without observing the data $(D)$; $p(D|\theta )$ is called likelihood, i.e., the probability that data $(D)$ are generated by the model with parameter value $\theta$; $p\left(D\right)$ is called evidence (or marginal likelihood), i.e., the overall probability of the data according to the model, determined by averaging across all possible parameters values weighted by the strength of belief in those parameters. Generally, researchers have knowledge about likelihood and prior distributions and want to estimate the posterior distribution, which represents an update of prior beliefs about parameters’ credibility once new information is available. One problem with this strategy is that, to obtain the posterior distribution of $\theta$, one has to compute the marginal likelihood, which in the case of continuous variables corresponds to estimating a very complicated integral, which could be impossible to solve analytically. A possible solution to this problem is to use the Markov Chain Monte Carlo (MCMC), i.e., a class of algorithms for sampling from a generic (unknown) probability distribution. To approximate the posterior distribution of interest, the MCMC strategy requires to know only the prior and the likelihood probability density functions, without evaluating the difficult integral at the denominator of the Bayes’ formula. In this paper, we decided to use the popular Gibbs algorithm to produce an approximation of the posterior distribution $p\left(\theta |D\right)$, by drawing a large sample of $\theta$ values. By applying the MCMC procedure with a number of simulations $S\to \infty$, the simulated posterior distribution converges to the real one with probability one. However, we need some diagnostic tools to assess the representativeness, accuracy, and efficacy of the MCMC procedure. In this sense, we will present graphical evidence using standard diagnostic tools like the trace plot, the density plot, the Gelman–Rubin statistics, and the chain’s autocorrelation functions. Trace plot and the Gelman–Rubin statistics (i.e., shrink factor) allow the investigation of the MCMC representativeness and convergence. The former depicts multiple superimposed chains, which, if overlapped, imply the representation of the same posterior distribution. The latter quantitatively assesses the convergence of multiple chains comparing the within-chain variability to the between-chain variability; if the chains have converged, the within-chain variability should be similar to the between-chain variability, resulting in a value close to 1. A value greater than 1 suggests lack of convergence, indicating that the chains have not yet adequately explored the target distribution. MCMC accuracy was tested using a measure of autocorrelation reporting the Effective Sample Size (ESS). The ESS is a metric that takes into account the autocorrelation within the MCMC chain and provides an estimate of the effective number of independent samples. It represents the number of uncorrelated samples that would contain the same amount of information as the original autocorrelated samples. An ESS amounting to 10.000 is generally recommended for stable 95% higher density intervals.

### Model inputs

Probabilities of diagnosis were obtained by the medical records of the patients enrolled in the clinical and research programs of the pediatric hospital. The probability of having a change in the clinical management was instead retrieved by the scientific literature. Change in clinical management refers to alterations or adjustments made in the medical care and treatment of a patient based on the results of a genetic test. This change is typically driven by the information obtained from the genetic test, which can provide valuable insights into an individual’s genetic makeup and its potential impact on their health.

The model included only direct costs according to the chosen perspective. All investigations, procedures, and outpatient assessments were retrospectively collected and revised by experienced pediatricians and geneticists, based on the pediatric hospital informative system. Exclusively diagnostic evaluations/procedures as well as management and therapeutic procedures’ costs were retained (i.e., costs of taking charge of patients). These cost items were estimated considering the different amount and type of consumables used, the diagnostic procedures performed before genetic/genomic tests, the personnel required and their working hours dedicated to the clinical discussion of each case (i.e., WES and WGS cases required wider multidisciplinary assessments).

Costs of testing were estimated according to the Italian NHS tariffs. The differences across these costs are due to the cost of consumables and the number of samples that can be analyzed simultaneously with the same equipment (e.g., sequencing chip). Specifically, the same analysis chip can be used for 96 samples in the case of SOC analysis, 24 samples in the case of WES analysis, and a single sample in the case of WGS analysis. Furthermore, there is a difference in the costs of data analysis, which was quantified economically as personnel costs (i.e., man-hours for each analysis). In conclusion, another driver of difference concerns the training costs of the staff involved for the genomic analysis. After -WES or -WGS testing costs were obtained from the scientific literature. Using historical foreign exchange rates and the Consumer Price Index, we expressed costs in 2022 Euros.

Utilities were not adopted since quality-adjusted life-year (QALY) estimation requires specific data seldom available for genomic technologies. Of note, international agencies [17, 18] recommend the use of QALY, when possible, since, being a standardized measure, eases the broad comparison of medical technologies and the consequent allocation of resources.

Notwithstanding, QALY is characterized by several pitfalls and usually is not used in the economic evaluation of genomic technologies [19]. In fact, it does not account for non-health related outcomes such as personal utility (e.g., increased sense of control, better self-awareness, and future planning) and family spillover effects [20–22]. In addition, estimating QALYs gained through genome-wide sequencing is challenging because it does not directly impact long-term outcomes but rather affects subsequent clinical management. Moreover, the patient population and care pathways are highly diverse, making it difficult to determine if early diagnosis leads to better health outcomes or longer survival at the population level. Diagnostic yield was used instead of QALYs as main outcome parameter since it is the most common [10, 12] outcome measure in clinical and economic studies of genome-wide sequencing [15, 23]. This choice was also supported by the lack of robust and reliable data to compute QALYs from changes in clinical management [24].

The study did not include the effectiveness outcomes related to parents or other family members, although the cost of WES and WGS did cover standard confirmatory testing and trio testing with biological parents. Table 1 lists the parameters, for each study arm, adopted in the base-case analysis.

**Table 1:** Summary of intervention-specific parameters adopted in the economic model

Model parameters	Base estimate	Distribution	References
Probability of diagnosis following testing strategy			
First-line SOC	0.43^a^	Beta	Hospital administrative data
First-line WES	0.58^a^	Beta	Hospital administrative data
First-line WGS	0.64^a^	Beta	Hospital administrative data
Change in clinical management SOC	0.06	Beta	[10]
Change in clinical management WES	0.17	Beta	[10]
Change in clinical management WGS	0.27	Beta	[10]
Testing costs			
Taking charge SOC	€29,870^b^	Log-normal	Hospital administrative data
Taking charge WES	€61,704^b^	Log-normal	Hospital administrative data
Taking charge WGS	€79,170^b^	Log-normal	Hospital administrative data
SOC testing	€450^c^	Log-normal	Hospital administrative data
WES testing	€1,800^c^	Log-normal	Hospital administrative data
WGS testing	€3,700^c^	Log-normal	Hospital administrative data
After WES or WGS, testing costs with Dx	€92	Log-normal	[13]
After WES or WGS, testing costs without Dx	€162	Log-normal	[13]
Diagnostic odyssey	€2,375	Log-normal	[25]

^a^Diagnostic yield for SOC, WES, and WGS was computed on a cohort of 300, 480, and 90 pediatric patients, respectively

^b^Costs include those accrued for consumables, personnel, and diagnostic investigations

^c^Costs associated with testing strategies are assumed to include labor, supplies, bioinformatics, and equipment

All estimates are computed on an annual basis. Log-normal distributions are specified by lower and upper limits of the 95% confidence intervals

*Dx* diagnosis, *WES* whole exome sequencing, *WGS* whole genome sequencing

### Model outcomes

The main outcomes of the model include the number of diagnoses and the expected costs. To estimate the expected cost for each decision tree arm, each cost is multiplied by the amount of resources required for the patient during the time in a particular health state and study arm.

### Model analyses

To address uncertainty regarding the estimated mean outcomes, a probabilistic sensitivity analysis (PSA) was conducted. Each parameter was assigned a specific distribution based on its characteristics. For diagnostic yields and change in clinical management, a beta distribution was used. The costs were parameterized by a log-normal distribution.

Table 1 shows the expanded list of model parameters with ranges used in the probabilistic sensitivity analyses.

For the PSA, 100,000 MCMC simulations were run using the Gibbs algorithm. The PSA involved randomly drawing sets of parameter values from probability distributions associated with each model parameter, and calculating incremental costs, incremental effectiveness, and ICERs for each set. The results were plotted using a cost-effectiveness plane (CEP), cost-effectiveness acceptability curves (CEAC), and the cost-effectiveness acceptability frontier (CEAF), as recommended by the International Society for Pharmacoeconomics and Outcomes Research (ISPOR) guideline [26] and the Second Panel on Cost-effectiveness in Health and Medicine [27]. The CEAC illustrates the probability that each intervention would be considered the optimal choice at various thresholds, while the CEAF displays the net monetary benefit at each willingness-to-pay (WTP) level and the level of uncertainty surrounding the optimal choice.

Expected incremental benefits (EIB) were also computed for WGS against the other testing strategies. The EIB shows the expected average incremental benefit by estimating the average incremental benefit of each simulation. The incremental benefit function can be modeled as a function of the WTP *k*:

$$IB(\theta ) = k\Delta_e - \Delta_c$$

Using the set of posterior samples S, the EIB is approximated as follows by

$$\frac{1}{S}{\sum }_{s}^{S}IB\left({\theta }_{s}\right),$$

where ${\theta }_{s}$ is the realized configuration of the parameters $\theta$ in correspondence of the s-th simulation. For the intervention of interest to be cost-effective, the EIB should be greater than 0, taking the WTP into account.

In addition, the Expected Value of Perfect Information (EVPI) and the Expected Value of Partially Perfect Information (EVPPI) were calculated to determine the value of collecting further information. The EVPI is computed for a specific threshold by calculating the difference between the expected value with perfect information (i.e., no uncertainty in model parameters) and the expected value with current information (i.e., uncertainty in model parameters [28]).

It is a measure to translate uncertainty associated with the cost-effectiveness evaluation in the model into an economic quantity. It is based on the Opportunity Loss (OL) (OL and OL = U_*_ − U_τ_), a measure of the potential losses caused by choosing the most cost-effective intervention on average when it does not result in the intervention with max utility in each simulation. U_*_ is the utility level associated to the best intervention in simulation S. U_τ_ is the utility level associated to the intervention preferred on average in simulation S. In this analysis, EVPI was used to estimate the value of future research to reduce or eliminate uncertainty in the cost-effectiveness of WGS compared to the other strategies in pediatric patients. The Expected Value of Partial Perfection Information (EVPPI) was also calculated to determine the value of reducing uncertainty in specific model parameters and identify which parameters were most important for the estimation of cost-effectiveness, thus guiding further research in those areas requiring additional information. The information-rank plot was also charted. For each parameter and value of the willingness-to-pay threshold, a bar chart is plotted to describe the ratio of EVPPI to EVPI, representing the relative importance of each parameter in terms of expected value of information.

The study was conducted according to the Consolidated Health Economic Evaluation Reporting Standards (CHEERS) Statement [29], and all analyses were carried out using R software (R Development Core Team).

## Results

### Base-case analysis

SOC testing had both the lowest expected costs and the lowest expected diagnostic yield for the investigated population.

From the NHS perspective, the base-case findings highlighted that, compared with SOC, first-line WES, and second-line WES, using first-line WGS yields an incremental cost of €25,072, €32,086, and €44,754 per added diagnosis, respectively.

First-line WGS was not cost-effective against second-line WGS being the ICER (i.e., €67,118 per added diagnosis) higher than the threshold.

Table 2 reports the mean discounted costs and outcomes as well as the base-case results for the simulated cohort.

**Table 2:** Base-case results over lifetime horizon

Strategy	Costs (€) (95% CI)	Δ costs (€)	Eff (95% CI)	Δ Eff	ICER	NMB (€)
WGS	898,703,504 (895,703,109– 899,703,898)	–	39,660 (37,506– 43,815)	–	Ref.	–
SOC	370,492,122 (369,491,97– 371,492,275)	52,821	18,592 (12,438– 20,747)	2.11	25,072	52,679
WES	705,518,737 (704,518,437– 707,519,036)	19,318	33,639 (31,485– 35,794)	0.60	32,086	10,682
SOC + WES	423,002,973 (417,002,819– 426,003,128)	47,570	29,031 (27,877– 34,186)	1.06	44,754	5,430
SOC + WGS	438,705,402 (433,705,247– 445,705,557)	45,999	32,448 (30,293– 36,603)	0.72	63,779	− 9,999

*CI* confidence intervals, *eff.* effectiveness, *ICER* incremental cost-effectiveness ratio, *NMB*, net monetary benefit

### Sensitivity analysis

**Fig. 2:**
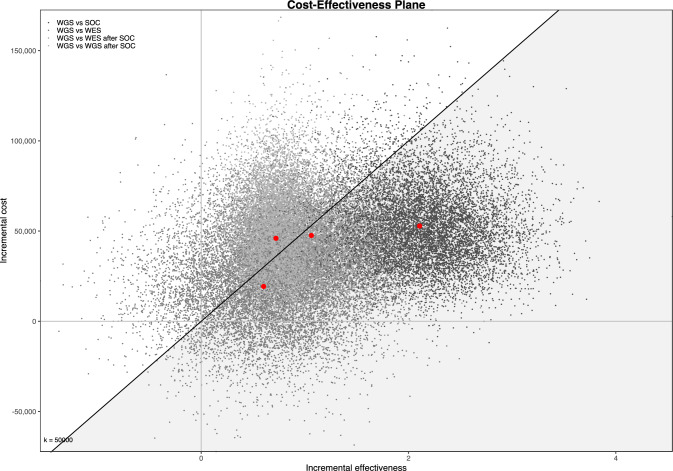
Cost-effectiveness plane from probabilistic sensitivity analysis

As shown in the cost-effectiveness plan (Fig. 2), the PSA confirmed the robustness of the base-case results at a threshold ranging from €30,000 to €50,000 per added diagnosis.

Contour plots, provided in supplementary materials (Figs. S1–S4) further highlighted that 66.9% to 99.6% of simulated points lie in the north-east quadrant of the cost-effectiveness plane, in which WGS generates more health gains but is more expensive than other testing strategies.

The CEAC and the CEAF, depicted in Figs. S5 and S6, showed that SOC had the highest probability of being cost-effective for a WTP threshold lower than €2,400 per added diagnosis, while second-line WES for a WTP threshold between €2,400 and €5,400 per added diagnosis. For all WTP levels above €5,400/diagnosis, tested up to €50,000/diagnosis, first-line WGS versus SOC strategy had the highest probability of being cost-effective (i.e., 94.1%), followed by first-line WGS versus first-line WES (i.e., 61.5%) and first-line WGS versus second-line WES (i.e., 57.6%).

All the values of the EIB were positive for all the testing strategies, particularly amounting to 52,519 for first-line WGS versus SOC, 10,786 for first-line WGS versus first-line WES, and 5,576 for first-line WGS versus second-line WES, with the exception of first-line WGS versus second-line WGS, which showed a negative EIB (Fig. S7).

### Value of information analysis

**Fig. 3:**
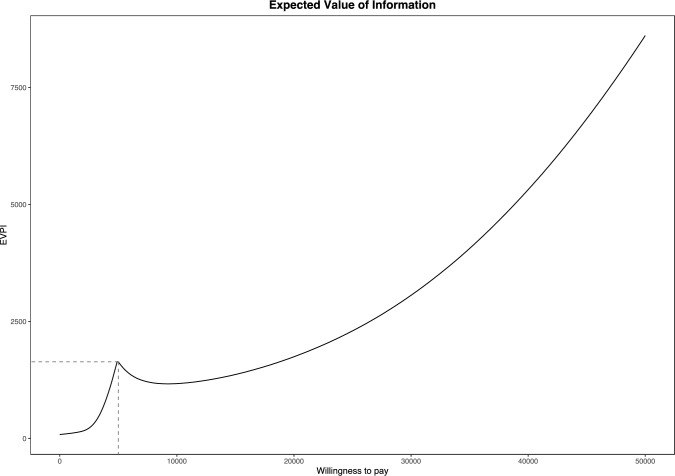
Population Expected Value of Perfect Information (EVPI) curve

From the PSA simulation, the EVPI per patient was estimated to be €8,610 on a WTP threshold of €50,000/diagnosis (Fig. X6). The EVPI decreased with a lower WTP threshold, amounting to €3,060 on a WTP level of €30,000/diagnosis (Fig. 3).

Additional examination of the EVPPI is shown in supplementary materials.

EVPPI, for a subset of parameters (i.e., transition probabilities), amounted to €594.8 (Fig. S8). Furthermore, as shown in Fig. S9, the cost of taking charge patients related to WGS investigations reported the highest ratio, showing how large is the expected value of gaining more information for this parameter.

### MCMC performance

The MCMC analysis was assessed using various diagnostic measures. Table S1, in supplementary materials, lists the summary statistics for the analysis. Trace plots showed that the chains for the sampled parameters exhibited stable behavior and overlapped, indicating convergence. Figures S10 to S18, in supplementary materials, depicts the trace plots for each cost parameters. Density plots illustrated smooth and unimodal distributions, suggesting well-behaved posterior distributions (Figs. S10 to S18).

The Gelman–Rubin statistics resulted in R-hat values close to 1 for all the parameters, further supporting the convergence of the chains (Table S1). In addition, ESS revealed values relative to the total number of samples close to 10.000 for most of the parameters, indicating efficient sampling and reliable estimation of the posterior distribution (Table S1).

Collectively, these diagnostic measures indicated that the MCMC algorithm performed well, with converged chains, high effective sample sizes, and well-estimated posterior distributions, providing confidence in the validity and reliability of the obtained results.

## Discussion

We assessed the cost-effectiveness of WGS considering 870 patients with suspected genetic disorders derived from the enrolled cohort of a large pediatric hospital in Italy. The present analysis showed that implementing first-line WGS would be a cost-effective strategy, against the majority of the other tested alternatives at a threshold of €30,000–50,000, for diagnosing infants with suspected genetic disorders. According to the sensitivity analyses, the findings were robust to most assumption and parameter uncertainty.

This study is contributing to the evidence base of cost-effectiveness specifically comparing WGS and WES providing evidence from an European health system not yet well represented in the scientific literature (i.e., Italy). Our results are consistent with those of Lavelle et al., focusing on WGS and WES in infants and children with rare and undiagnosed diseases, which showed the cost-effectiveness of first-line WGS strategy for the target populations with an ICER of, respectively, $15,048 and $27,349 per diagnosis [13]. Present data broadly support also the study of Incerti et al. in the United States, demonstrating the cost-effectiveness and cost-saving of WGS as a first-line diagnostic tool for children and infants with suspected genetic disorders. Although these authors adopted a different modeling approach, the cost-effectiveness of WGS (i.e., ICER of $15,904) is in line with our results [14].

Another study investigated the economic benefits of WGS versus WES, even though not using modeling tools, confirming that WGS is the optimal genomic test choice for maximal diagnosis in Mendelian disorders [30].

Moreover, as shown by comprehensive systematic reviews and meta-analyses, WGS significantly improves both accuracy and economic aspects in pediatric patients with suspected genetic disorders. In particular, Clarke et al. estimated that the pooled diagnostic yield of WGS was higher than the diagnostic yield of WES and usual care [10] while Nurchis et al. showed the cost-effectiveness of WGS over WES by pooling their incremental net benefits [31].

The current study comes at an important time considering the recent recommendations of the American College of Medical Genetics to adopt WES and WGS as tools for diagnosing genetic conditions in children [32]. Notwithstanding, Italy trails behind other countries concerning the reimbursement for genomic sequencing, especially WGS. Nowadays, the adoption of WGS has been more limited, with respect to WES, due to higher costs and still limited gains in terms of clinical benefit [12]. Particularly, evidence in the scientific literature showed that capital, maintenance and storage costs of WGS are higher than those associated with WES, contributing to limit the widespread adoption of WGS.

The principal finding of this analysis provides insights for a main implication suggesting that first-line WGS allows often an earlier and precise diagnosis. From this perspective, implementing WGS as a first-tier strategy has the potential to bend the cost trajectory of diagnosing and managing children with suspected genetic disorders [33–35]. The study findings also raise policy implications for WGS reimbursement in Italy. Following the recent guidelines released by international supranational organizations [32, 36] and recently published evidence, policy-makers should define a tailored diagnosis-related group (DRG) tariff for the reimbursement of the inpatient and outpatient health services related to this diagnostic test. Developing sound genomic policies and specific reimbursement tariffs is of paramount importance for guaranteeing healthcare sustainability by applying the three core functions of Public Health [37] (i.e., assessment, policy development, and assurance) to the delivery of WGS within the health care services.

Furthermore, beyond informing treatment decisions, receiving a genetic diagnosis could significantly impact patients and their families on psychosocial levels, alleviating uncertainty and anxiety, thereby potentially enhancing overall well-being. Cascade testing within families, initiated by a genetic diagnosis, can lead to early interventions and preventive measures, which in the long term may result in substantial cost savings by averting disease progression and related health care expenses. In addition, genetic diagnoses contribute to research and therapeutic development, as identified mutations may become targets for drug development and personalized treatment strategies, offering hope for improved patient outcomes and potentially reducing the overall economic burden of the condition.

The analysis presented herein has several limitations and strengths. First, effectiveness was not parametrized as QALYs, but as clinical outcomes, such as the number of molecular diagnoses and active treatment changes. Given the lack of a clear established threshold for budget allocation related to the outcomes, it could be challenging to interpret the cost-effectiveness findings and compare them with other economic analyses of health technologies. Nonetheless, in line with available economic studies, we assumed that the threshold that the society is willing to pay for a single QALY is the same amount of money which is willing to pay for one more diagnosis [38–41]. Using the point estimate for the change in clinical management parameter, as retrieved by the scientific literature, may overestimate the benefits of WGS versus WES given the overlapping confidence intervals reported in the meta-analysis. However, this may represent a bias for the secondary study itself. At the moment, it represents the most updated published reference suitable with our outpatient cohort. Increasing evidence indicates that WES data re-analysis, taking into account new knowledge, has been demonstrated to significantly increase the diagnostic yield [42]. The used Bayesian model, however, did not take into account re-analysis because this is not yet a regular practice in Italy [43], even though, in Australia, re-analysis is included in the reimbursement description of the genomic test [44, 45]. This represents a limitation of our assessment as the inclusion of re-analysis would lead to an increase in costs of WES and WGS with respect to standard practices, but potentially cost-effective compared to single-analysis WES or WGS. This is given by lower cost of re-analysis, and as more is learned about causal variants in this patient’s population, it is likely that more diagnoses will be identified. Furthermore, model inputs did not include capital, maintenance, and storage costs. Van Nimwegen et al. [46] found that these costs are higher for WGS than WES, thus limiting the benefits of WGS over WES. Furthermore, we did not consider the possibility that WGS or WES might uncover incidental findings in the child or in a parent. If the benefits of returning incidental findings were factored in, the estimated cost-effectiveness of WES and WGS would likely improve, as previous research, conducted in the USA, has shown that while returning incidental findings leads to increased costs, it also enhances health benefits [47]. Nevertheless, in publicly funded health systems, there is still scarce evidence for clinical benefits from returning incidental findings [48]. There is still a debate among experts in pediatric genetics whether incidental findings in children concerning adult-onset conditions should be disclosed to parents [49]. Patient selection (i.e., focusing only on outpatients) may have influenced diagnostic yields, thus resulting in a structural limitation of the study. However, it is worth mentioning that, in Italy, there are currently no specific national guidelines on patient selection criteria (i.e., whether offering WGS or WES as opposed to SOC to a patient) neither for pediatric nor for adult population.

Given the lack of primary data, we assumed that changes in clinical management occur only in the “diagnosis” arm of the decision tree. This represents a model limitation since other studies demonstrated that WES or WGS may have clinical utility even in patients not receiving a diagnosis [50].

An additional caveat was the lack of primary data for some cost assumptions, from Italy. Therefore, we had to use estimates from Canada and United States. Notwithstanding, it is commonly believed that the United States has double healthcare costs despite presenting comparable utilization rates to other high-income countries due to different practices in rationing services and prices of labor, pharmaceuticals, and administrative costs [51]. Another limitation is unavailability of data that could be used to estimate the societal value of detecting RDs. Recent guidelines have increasingly recognized the significance of societal costs. In 2020, the Institute for Clinical and Economics Review emphasized the need for a societal co-base case accounting for family caregiver costs and productivity impacts, given their substantial influence on the estimated cost-effectiveness. The Second Panel on Cost Effectiveness in Health and Medicine has also called for a dual base case, with one analysis taking into account societal impacts. In the near future, incorporating societal costs and benefits in cost-effectiveness analyses will provide more favorable estimates of value for WES and WGS because rare and undiagnosed diseases place considerable burdens on families and lead to productivity losses. In line with the seminal study of Wu et al. [52] focused on WES, further studies should investigate the broader consequences of WGS in children with suspected genetic disorders and their relatives, including family spillover effects, healthcare costs, and productivity costs. Other cost-effectiveness analyses should be conducted including additional input parameters on maintenance and capital costs and re-analyses procedures.

## Conclusion

NGS-based genome-wide diagnostic methods are becoming more prevalent in the clinical settings. The findings provided a cost-effectiveness model showing that WGS is, indeed, a cost-effective strategy when the model assumption holds true. Strong clinical and economic evidence is needed to justify implementation of WGS to demonstrate its advantages. Lessons learnt from this modeling study reinforces the adoption of first-tier WGS, as a cost-effective strategy, depending on actual difficulties for the Italian NHS to properly allocate limited resources. Of note, this paper tried to fill a gap in favor of implementing WGS in a health system (i.e., Italy) where there is very little prior evidence.

### Supplementary Information

Below is the link to the electronic supplementary material.

Supplementary file1 (DOCX 10113 KB)

## Data Availability

The datasets generated during and/or analyzed during the current study are available from the corresponding author on reasonable request.
