# Supplementary material for: Bayesian cost-effectiveness analysis of Whole genome sequencing versus Whole exome sequencing in a pediatric population with suspected genetic disorders
Source: Eur J Health Econ. 2023 Nov 17;25(6):999–1011. doi: 10.1007/s10198-023-01644-0 (PMC11283423; doi:10.1007/s10198-023-01644-0)
Supplement: Supplementary file 1 — Supplementary file1 (DOCX 10113 KB) [file 10198_2023_1644_MOESM1_ESM.docx]

**Bayesian cost-effectiveness of whole genome sequencing versus whole exome sequencing for the paediatric population with suspected genetic disorders**

**Supplementary Materials**

**Figure S1.** Contour plot for WGS versus SOC

**Figure S2.** Contour plot for WGS versus WES

**Figure S3.** Contour plot for WGS versus second-line WES

**Figure S4.** Contour plot for WGS versus second-line WGS

**Figure S5.** Cost-effectiveness Acceptability Curve (CEAC)

**Figure S6.** Cost-effectiveness Acceptability Frontier Curve (CEAF)

**Figure S7.** Expected Incremental Benefit (EIB) for each testing strategy

**Figure S8.** Population Expected Value of Partially Perfect Information (EVPPI) curve

**Figure S9.** Information-rank plot reporting a ranking of the model parameters in terms of their impact on the expected value of information

**Figure S10.** Convergence diagnostics for the output of JAGS related to the cost parameter “taking charge SOC”

**Figure S11.** Convergence diagnostics for the output of JAGS related to the cost parameter “taking charge WES”

**Figure S12.** Convergence diagnostics for the output of JAGS related to the cost parameter “taking charge WGS”

**Figure S13.** Convergence diagnostics for the output of JAGS related to the cost parameter “cost of SOC testing”

**Figure S14.** Convergence diagnostics for the output of JAGS related to the cost parameter “cost of WES testing”

**Figure S15.** Convergence diagnostics for the output of JAGS related to the cost parameter “cost of WGS testing”

**Figure S16.** Convergence diagnostics for the output of JAGS related to the cost parameter “cost of diagnostic odyssey”

**Figure S17.** Convergence diagnostics for the output of JAGS related to the cost parameter “After WGS/WES testing costs with diagnosis”

**Figure S18.** Convergence diagnostics for the output of JAGS related to the cost parameter “After WGS/WES testing costs without diagnosis”

**Table S1.** Summary statistics of the marginal posterior distribution for each of the model parameters.

| **Parameters** | **Mean** | **SD** | **Lower bound (2.5%)** | **Upper bound**  **(97.5%)** | **R-hat** | **N.eff** |
| --- | --- | --- | --- | --- | --- | --- |
| beta.c.soc | 0.061 | 0.006 | 0.05 | 0.072 | 1.001 | 30000 |
| beta.c.wes | 0.176 | 0.037 | 0.11 | 0.254 | 1.001 | 30000 |
| beta.c.wgs | 0.28 | 0.069 | 0.156 | 0.423 | 1.001 | 22000 |
| beta.soc | 0.431 | 0.03 | 0.372 | 0.49 | 1.001 | 24000 |
| beta.wes | 0.579 | 0.031 | 0.518 | 0.64 | 1.001 | 30000 |
| beta.wgs | 0.629 | 0.031 | 0.566 | 0.688 | 1.001 | 16000 |
| dx.c.soc | 26.067 | 5.923 | 15 | 38 | 1.001 | 30000 |
| dx.c.soc.wes | 58.23 | 14.91 | 32 | 91 | 1.001 | 30000 |
| dx.c.soc.wes.int | 26.067 | 5.923 | 15 | 38 | 1.001 | 30000 |
| dx.c.soc.wgs | 100.136 | 27.347 | 52 | 158 | 1.001 | 30000 |
| dx.c.soc.wgs.int | 26.067 | 5.923 | 15 | 38 | 1.001 | 30000 |
| dx.c.wes | 102.271 | 23.997 | 60 | 154 | 1.001 | 19000 |
| dx.c.wgs | 175.794 | 45.773 | 94 | 273 | 1.001 | 30000 |
| dx.soc.wes | 329.786 | 29.102 | 274 | 388 | 1.001 | 30000 |
| dx.soc.wes.int | 430.755 | 33.834 | 365 | 498 | 1.001 | 30000 |
| dx.soc.wgs | 357.799 | 30.119 | 300 | 418 | 1.001 | 15000 |
| dx.soc.wgs.int | 430.755 | 33.834 | 365 | 498 | 1.001 | 30000 |
| dx.soc_i | 430.755 | 33.834 | 365 | 498 | 1.001 | 30000 |
| dx.wes_i | 579.394 | 34.604 | 511 | 647 | 1.001 | 30000 |
| dx.wgs_i | 628.524 | 34.773 | 559 | 695 | 1.001 | 20000 |
| no.dx.c.soc | 404.688 | 32.243 | 342 | 468 | 1.001 | 30000 |
| no.dx.c.soc.wes | 271.556 | 27.711 | 218 | 327 | 1.001 | 30000 |
| no.dx.c.soc.wes.int | 404.688 | 32.243 | 342 | 468 | 1.001 | 30000 |
| no.dx.c.soc.wgs | 257.664 | 33.925 | 192 | 325 | 1.001 | 15000 |
| no.dx.c.soc.wgs.int | 404.688 | 32.243 | 342 | 468 | 1.001 | 30000 |
| no.dx.c.wes | 477.123 | 36.803 | 405 | 549 | 1.001 | 30000 |
| no.dx.c.wgs | 452.73 | 51.396 | 350 | 550 | 1.001 | 14000 |
| no.dx.soc | 569.245 | 33.834 | 502 | 635 | 1.001 | 30000 |
| no.dx.soc.wes | 670.214 | 29.102 | 612 | 726 | 1.001 | 30000 |
| no.dx.soc.wgs | 642.201 | 30.119 | 582 | 700 | 1.001 | 13000 |
| no.dx.wes | 420.606 | 34.604 | 353 | 489 | 1.001 | 28000 |
| no.dx.wgs | 371.476 | 34.773 | 305 | 441 | 1.001 | 19000 |
| psi[1] | 29879.9 | 7504.5 | 17802.4 | 46877.6 | 1.001 | 30000 |
| psi[2] | 61544.1 | 15459.1 | 36805.1 | 96994.8 | 1.001 | 26000 |
| psi[3] | 79118.253 | 19913 | 47377.1 | 125374.7 | 1.001 | 11000 |
| psi[4] | 449.707 | 112.589 | 269.4 | 705.9 | 1.001 | 14000 |
| psi[5] | 1801.119 | 451.5 | 1071.9 | 2824.4 | 1.001 | 30000 |
| psi[6] | 3696.482 | 933.164 | 2206.9 | 5827.8 | 1.001 | 30000 |
| psi[7] | 2375.061 | 595.447 | 1426.2 | 3745.9 | 1.001 | 30000 |
| psi[8] | 91.499 | 22.802 | 54.842 | 144.1 | 1.001 | 20000 |
| psi[9] | 162.833 | 40.815 | 97.667 | 257.2 | 1.001 | 30000 |

The range between the 2.5% and 97.5% quantiles is the 95% credible interval for each parameter, the Bayesian equivalent of a confidence interval.

The last two columns in the table are convergence diagnostics. The effective sample size (n.eff) is a number smaller than or equal to the number of samples saved from the chains (3 * (12000 - 2000) / 10). The higher the autocorrelation in the saved samples, the smaller the effective sample size. R-hat is the potential scale reduction factor measuring how well the Markov chains have mixed and should ideally have a value very close to 1.
